# Long-Term Particulate Matter (PM) Exposure Promotes Non-Small-Cell Lung Cancer (NSCLC) Angiogenesis Through Up-Regulation of VEGFA

**DOI:** 10.3390/cancers17172868

**Published:** 2025-08-31

**Authors:** Khaled Omran, Ya-Jing Jiang, Trung-Loc Ho, Iqra Kousar, Chih-Hsin Tang, Ming Tan

**Affiliations:** 1 Graduate Institute of Biomedical Sciences, China Medical University, Taichung 406040, Taiwan; u112305107@cmu.edu.tw (K.O.); u110305105@cmu.edu.tw (T.-L.H.); u113305110@cmu.edu.tw (I.K.);; 2 Oncology Department, Tobruk Medical Centre, Tobruk 3WFJ-W5X, Libya; 3 Department of Medicine, Faculty of Medicine, Tobruk University, Tobruk 3VGW-F8, Libya; 4 Department of Pharmacology, School of Medicine, China Medical University, Taichung 406040, Taiwan; 5 Chinese Medicine Research Center, China Medical University, Taichung 406040, Taiwan; 6 Department of Medical Laboratory Science and Biotechnology, College of Medical and Health Science, Asia University, Taichung 406040, Taiwan; 7 Institute of Biochemistry and Molecular Biology, China Medical University, Taichung 406040, Taiwan; 8 Cancer Biology and Precision Therapeutics Center, China Medical University, Taichung 406040, Taiwan

**Keywords:** NSCLC, air pollution, PM, angiogenesis, metastatic potential, VEGFA, MAPK/ERK

## Abstract

Background: LUAD, the most common subtype of lung cancer, particularly in non-smokers, is significantly influenced by air pollution from fine particulate matter (PM). One suspected method by which PM contributes to cancer progression is through angiogenesis, which promotes tumor growth and metastasis. This study was conducted to explore the impact of long-term PM exposure on the progression of LUAD, focusing on angiogenesis promotion. Methods: We conducted an integrative bioinformatics analysis incorporating epidemiological and transcriptomic datasets from public repositories (TCGA and GEO) to evaluate differential VEGFA expression in LUAD tissues and its relationship to regional PM exposure. In vitro and in vivo assays using PM-adapted NSCLC cell lines and murine xenograft models served as secondary confirmatory experiments supporting the computational results. Results: Epidemiological analysis revealed a strong positive correlation between long-term PM exposure and lung adenocarcinoma mortality across U.S. states (r = 0.7638, *p* < 0.0001), underscoring a population-level impact. Bioinformatics analysis identified a significant upregulation of VEGFA in NSCLC tumors from regions with high PM levels, with VEGFA overexpression also associated with poorer patient survival. Gene ontology and pathway enrichment analyses implicated angiogenesis-related processes. These findings were supported by experimental models, in which long-term PM exposure on human and murine LUAD cell lines (A549, H1299, and LLC) induced VEGFA and p-ERK overexpression. Furthermore, PM-exposed cells enhanced angiogenesis processes, as evidenced by increased endothelial cell tube formation and migration in vitro, and promoted tumor vascularization in a xenograft model. These pro-angiogenesis effects were abrogated following inhibition of the MAPK signaling pathway or blockade of VEGFA. Conclusions: Our findings reveal a compelling molecular link between PM exposure and NSCLC progression, centered on VEGFA-driven angiogenesis and urging the need to reduce ambient PM exposure to mitigate its oncogenic impact.

## Simple Summary

This study uses various bioinformatics analyses to investigate how exposure to fine particulate matter (PM) in air pollution promotes the progression of lung adenocarcinoma (LUAD), particularly through its effects on tumor angiogenesis. Analyzing data from 47 U.S. states revealed a strong positive correlation between ambient PM levels and lung cancer mortality, emphasizing the public health impact of air pollution. Through integrative transcriptomic analyses, we identified VEGFA as a consistently upregulated angiogenic factor in LUAD patients exposed to high PM levels, and its overexpression was associated with poor survival outcomes. Pathway analysis implicated the MAPK/ERK signaling cascade could be a driver of this VEGFA upregulation. These computational findings were supported by in vitro and in vivo experiments, which demonstrated enhanced VEGFA-mediated angiogenesis following PM exposure. Overall, this study highlights a molecular mechanism linking air pollution to cancer progression and reinforces the importance of reducing environmental PM exposure as a cancer prevention measure.

## 1. Introduction

Despite advancements in drug development for lung cancer, particularly in immunotherapy, targeted therapy, and early detection techniques, lung cancer continues to be a leading cause of cancer-related deaths globally. It imposes a significant health burden, accounting for approximately one in eight (12.4%) cancer diagnoses worldwide and nearly one in five (18.7%) cancer-related deaths, according to the 2022 global cancer statistics (GLOBOCAN) [1,2,3,4]. Non-small-cell lung cancer (NSCLC) constitutes approximately 80% of all lung cancer cases, with adenocarcinoma (LUAD) being the most prevalent subtype of more than 40% of lung cancer and 60% of NSCLC [5,6,7]. Compared to lung cancer in ever-smokers, lung cancer in never-smokers is more common among women, individuals of East Asian ancestry, and younger age groups, with LUAD being the predominant histological subtype [8,9,10]. While tobacco smoking is a well-established risk factor for all lung cancer subtypes, air pollution, specifically exposure to particulate matter 2.5 μm (PM2.5), has emerged as a significant contributor to lung cancer development and progression [11,12,13,14,15,16,17].

PM is a diverse combination of very fine solid particles and liquid droplets suspended in the air, consisting of substances such as acids, organic compounds, metals, and particles originating from soil or dust. The size of PM is directly linked to its potential to cause health issues, with smaller particles posing a greater risk than larger ones. Consequently, PM2.5 has more severe adverse effects on health compared to PM10, especially in the context of air pollution [18]. PM2.5 encompasses a complex mixture of solid and liquid particles suspended in the air, with diameters less than 2.5 μm [11,12,13,19]. These fine particles can penetrate deep into the respiratory system, reaching the alveoli, where they can trigger a cascade of adverse effects [20,21,22]. The World Health Organization (WHO) emphasizes that both developed and developing nations exhibit evidence of airborne particulates and their detrimental consequences on public health [23,24].

Studies have consistently shown a strong association between PM exposure and lung cancer mortality [25,26,27,28]. For example, an ecological analysis found a significant positive association between long-term exposure to PM and lung cancer mortality rates [12]. A study reported that a 10 μg/m^3^ increase in PM exposure was associated with a 6.5% increase in the probability of a shorter survival time for lung cancer patients [17]. These findings underscore the urgent need to address PM pollution as a critical public health concern, especially concerning lung cancer.

The link between PM exposure and lung cancer is complicated, involving several key mechanisms. PM can induce chronic inflammation in the lungs, establishing a microenvironment that favors tumor development [29]. Moreover, PM components can generate reactive oxygen species, leading to oxidative stress, DNA damage, and uncontrolled cell proliferation, ultimately promoting tumorigenesis [30]. Emerging evidence suggests that PM can disrupt the delicate balance of the immune system, impairing immune surveillance and allowing cancer cells to evade immune destruction [20,31]. Additionally, exposure to PM can stimulate the formation of new blood vessels, a process called angiogenesis, which is crucial for tumor growth and metastasis [32,33].

Primarily, angiogenesis is a natural molecular process that is involved in various physiological events including reproduction, development, and tissue repair. However, it is exploited by tumor growth and metastasis [34]. It is widely accepted that angiogenesis is one of the hallmarks of cancer that is required by other hallmarks of cancer such as tumor invasion and metastasis. The mechanisms governing endothelial cell development, differentiation, and homeostasis as well as the concept of the angiogenesis switch are well understood. Over the past decades, additional signaling pathways involving endothelial cell receptors and growth factors have been identified. These pathways play crucial roles in regulating endothelial cell phenotypes in both developmental and tumor-associated angiogenesis, highlighting their complex regulation [35]. VEGFA, a crucial regulator of angiogenesis, promotes the growth of new blood vessels and enhances vascular permeability by binding to and activating its receptors on endothelial cells [32].

Further research has shed light on the molecular mechanisms through which PM promotes lung cancer progression. For instance, one study highlighted the activation of the AhR-TMPRSS2-IL18 pathway by PM, leading to enhanced tumor growth and invasiveness [36]. Another study found that PM exposure facilitates lung cancer proliferation through glutamine metabolism and the up-regulation of amphiregulin, a growth factor that promotes cell proliferation [37]. However, the impact of long-term exposure to PM on tumor angiogenesis as well as the role of this angiogenesis in the progression of PM-promoted lung cancer remains unclear. A better understanding of these factors could offer new insights for developing therapeutic strategies to treat or prevent lung cancer associated with PM exposure.

Given the global health burden of lung cancer and the mounting evidence linking PM exposure to lung cancer development, this study was conducted to examine the impact of long-term PM exposure on lung cancer, focusing on its role in promoting angiogenesis, a critical factor in disease progression and metastasis. We hypothesize that prolonged PM exposure promotes angiogenesis in LUAD via upregulation of VEGFA.

## 2. Materials and Methods

### 2.1. Materials

PM urban dust (Standard reference material 1649b) was purchased from the National Institute of Standards and Technology NIST (MD, USA). VEGFA (catalog number: SC-7269), VEGFB (catalog number: SC-80442), ANG1 (catalog number: SC-74528), PDGFA (catalog number: SC-9974), PDGFB (catalog number: SC-365805), FGF2 (catalog number: SC-74412), ERK (catalog number: SC-514302), p-ERK (catalog number: SC-7383), p-JNK (catalog number: SC-7345), and CD133 (catalog number: SC-365537) were purchased from Santa Cruz Biotechnology (Dallas, TX, USA). VEGFA neutralizing antibody (catalog number: ab9570) and CD31 (catalog number: (catalog number: ab28364) were purchased from Abcam (Cambridge, MA, USA). Β-Actin (catalog number: A6730) was purchased from Sigma-Aldrich (St. Louis, MO, USA).

All small interfering RNAs were purchased from Dharmacon (Lafayette, CO, USA). ERK inhibitor was from Sigma-Aldrich (St. Louis, MO, USA). VEGFA recombinant protein was from PeproTech (Rocky Hill, NJ, USA). Matrigel was from (BD Biosciences, Bedford, MA, USA). Human VEGFA ELISA DuoSet Human VEGF kit (Catalog Number: DY293B) was from (R&D Systems, Inc., Minneapolis, MN, USA). All primers were from Applied Biosystems (Foster City, CA, USA) (Table 1).

### 2.2. Cell Culture

The PM was dispersed in ultrapure water and sonicated for 30 min using an ultrasonic bath before each use. All PM samples were stored at 4 °C. Subsequently, A549 cells, H1299, and LLC (A549.Par, H1299-Par, and LLC-Par) were subjected to long-term (30–60 days) exposure to 12.5–25 μg/mL of PM, resulting in the A549-PM, H1299-PM, and LLC-PM cell lines. A549 and H1299 cell lines were cultured in Dulbecco’s Modified Eagle Medium (DMEM; Gibco, Grand Island, NY, USA), while LLC cells were cultured in Roswell Park Memorial Institute (RPMI) 1640. Both media were supplemented with 100 μg/mL streptomycin, 100 U/mL penicillin, and 10% fetal bovine serum (FBS; Gibco, USA). The cells were incubated at 37 °C in a humidified environment with 5% CO_2_. Endothelial progenitor cells (EPCs) were cultured and expanded in MV2 complete medium, which includes MV2 basal medium and growth supplement (PromoCell, Heidelberg, Germany), along with 20% defined fetal bovine serum (DFBS) (HyClone, Logan, UT, USA). The EPC cultures were seeded onto plasticware coated with 1% gelatine and maintained at 37 °C in a humidified atmosphere with 5% CO_2_.

### 2.3. Quantitative Real-Time Polymerase Chain Reaction (qPCR) Analysis

RNA was isolated from A549-Par and A549-PM cells using TRIzol^®^ reagent kit (Catalog No. 15596026) (MDBio Inc., Taipei, Taiwan). GE NanoVue Plus spectrophotometer (GE Healthcare Life Sciences, Pittsburgh, PA, USA) was used to determine RNA concentrations. M-MLV Reverse Transcriptase kit (Catalog No. 28-025-013) (Invitrogen, Thermo Fisher Scientific, Waltham, MA, USA) was used to synthesize Complementary DNA (cDNA). For real-time quantitative polymerase chain reaction (qPCR) analysis, SYBR™ Green Master Mix (Applied Biosystems, Waltham, MA, USA) was utilized.

### 2.4. Western Blot Analysis

Cell lysates were prepared using RIPA buffer supplemented with a protease inhibitor cocktail. Protein concentrations were measured using a BCA Protein Assay Kit (Thermo Fisher Scientific Inc., Rockford, IL, USA). The proteins were separated by SDS-PAGE and subsequently transferred onto Immobilon^®^ polyvinylidene difluoride (PVDF) membranes. The membranes were blocked with 5% BSA at room temperature for 1 h and then incubated with primary antibodies (diluted 1:1000) for another hour. After washing three times with TBST buffer, the blots were incubated with secondary antibodies. Protein bands were detected and visualized using the ImageQuant™ LAS 4000 system (GE Healthcare, Little Chalfont, UK).

### 2.5. Enzyme-Linked Immunosorbent Assay (ELISA) Assay

A549-PM cells were cultured with or without the transfection of siRNAs or the treatment with the ERK inhibitor. The conditioned medium (CM) from these cells and A549-Par were collected, and VEGF levels were quantified by a VEGF-A ELISA kit; the procedures of the manufacturer were followed.

### 2.6. Tube Formation Assay

Matrigel was thawed at 4 °C overnight. A 48-well plate was coated with 100 µL of Matrigel per well and incubated at 37 °C one hour to allow solidification. Endothelial progenitor cells (EPCs) were seeded at a density of 3 × 10^4^ cells per well in 200 µL of culture medium, consisting of a 1:1 mixture 10% DFBS MV2 medium and conditioned media (CM) of A549-Par and A549-PM with or without treatments. The plate was incubated at 37 °C for 6 h to allow tube formation. The resulting tubular structures were visualized and photographed at ×200 magnification using a light microscope. The number of tube branches and the total tube length were quantified using MacBiophotonics ImageJ software [38].

### 2.7. EPCs Migration Assay

CM from A549-Par and A549-PM cells, with or without treatment, was added to the lower chamber of a Boyden chamber (AP48 “microchemotaxis chamber,” Neuro Probe, Cabin John, MD, USA). Endothelial progenitor cells (EPCs) were harvested and seeded into the upper chamber at a density of 10^4^ cells/well in 10% DFBS MV2 medium. The chambers were incubated for 24 h at 37 °C to allow cell migration through the membrane pores into the lower compartment. After incubation, the cells were fixed with 3.7% formaldehyde solution for 15 min and stained with 0.05% crystal violet in PBS for another 15 min. Non-migrated cells on the upper side of the membrane were gently removed using cotton-tipped swabs and rinsed with PBS. Migrated cells on the underside of the filters were visualized under a microscope, counted, and quantified using MacBiophotonics ImageJ software [39].

### 2.8. Immunohistochemistry (IHC) Staining

Tumor tissue sections were deparaffinized using xylene and rehydrated in a graded concentrations of ethanol series. After antigen retrieval in boiling 10 mM sodium citrate, pH 6.0, for 12 min, the blocking of intrinsic peroxidase activity was carried out by incubation with 3% hydrogen peroxide. IHC staining was performed using the NovoLink Polymer System (Catalog No. RE7150-CE, Leica Microsystems, Wetzlar, Germany) following the manufacturer’s instructions. Human primary antibodies were applied to the slides and incubated overnight at 4 °C. Antibodies against VEGFA and CD133 were used at a dilution of 1:100, while the CD31 antibody was applied at a 1:50 dilution. A biotin-labeled secondary antibody was then added and incubated for 1 h at room temperature. Staining was visualized using 3,3′-diaminobenzidine tetrahydrochloride (DAB), and images were captured using a light microscope.

### 2.9. Bioinformatics and the GEO Dataset Analysis

Using the TCGA (The Cancer Genome Atlas) dataset for lung adenocarcinoma (LUAD), we analyzed VEGFA gene expression in 515 patients. Differential expression of the VEGFA gene between LUAD tissues and adjacent normal tissues was assessed using data available on TNMplot.com. Survival analysis was conducted on KMplot.com, focusing exclusively on LUAD patients. Additionally, variations in VEGFA gene expression between LUAD patients residing in regions with high versus low PM concentrations in China were analyzed using datasets from the NCBI database (accession numbers: GSE165298 and GSE268175). Further datasets were retrieved from NCBI (accession numbers: GSE89039, GSE220165, GSE220252, and GSE213590) to support the analysis. Gene ontology (GO) analysis was performed using the TNMplot.com platform to explore the functional annotations and biological pathways associated with VEGFA.

### 2.10. Statistical Analysis

All data are presented as the mean ± standard deviation (SD) or mean ± standard error of the mean (SEM), as indicated. Statistical differences between the two experimental groups were evaluated using the Student’s *t*-test. For comparisons involving more than two groups, one-way analysis of variance (ANOVA) was performed, followed by Bonferroni’s post hoc test for multiple comparisons. Pearson’s correlation test was used to assess the strength and direction of linear relationships between variables. In all analyses, a *p*-value of <0.05 was considered statistically significant.

## 3. Results

### 3.1. Elevated Levels of PM in Ambient Air Are Significantly Associated with an Increased Mortality Rate Among Lung Cancer Patients

**Figure 1:**
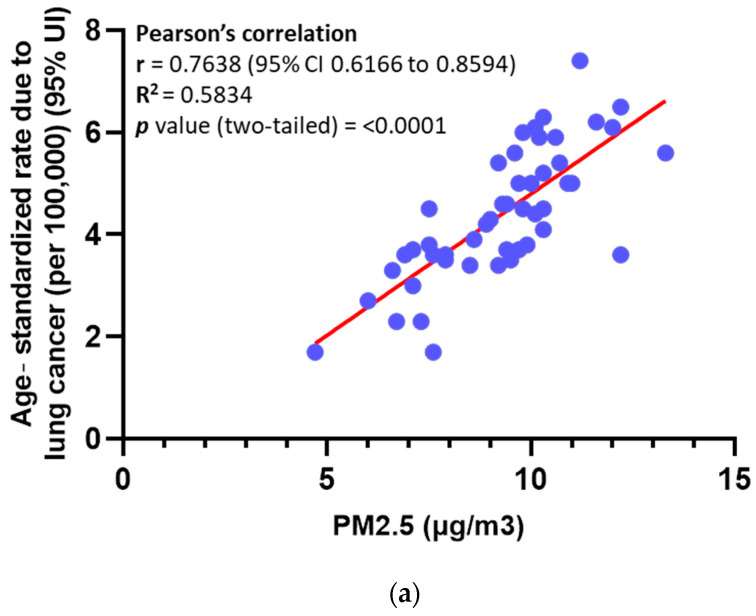

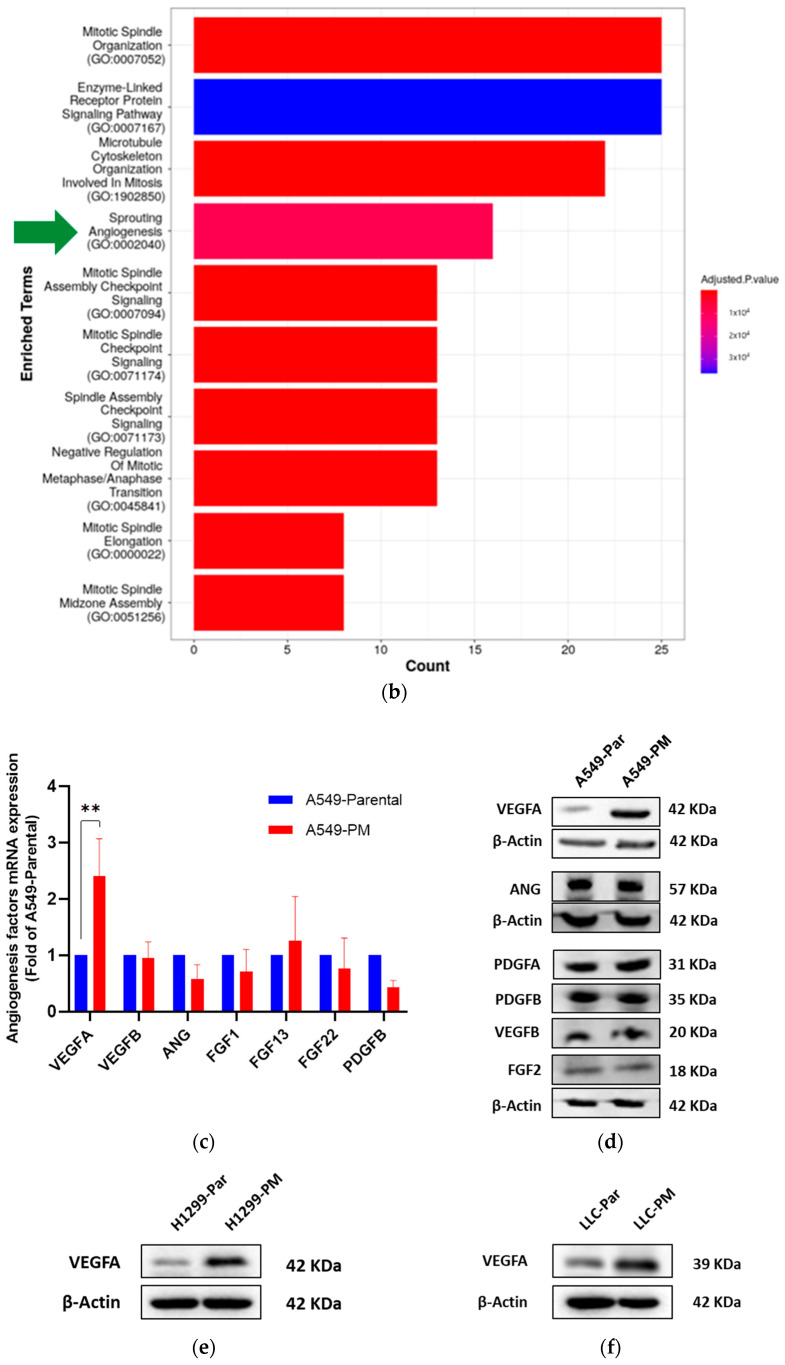

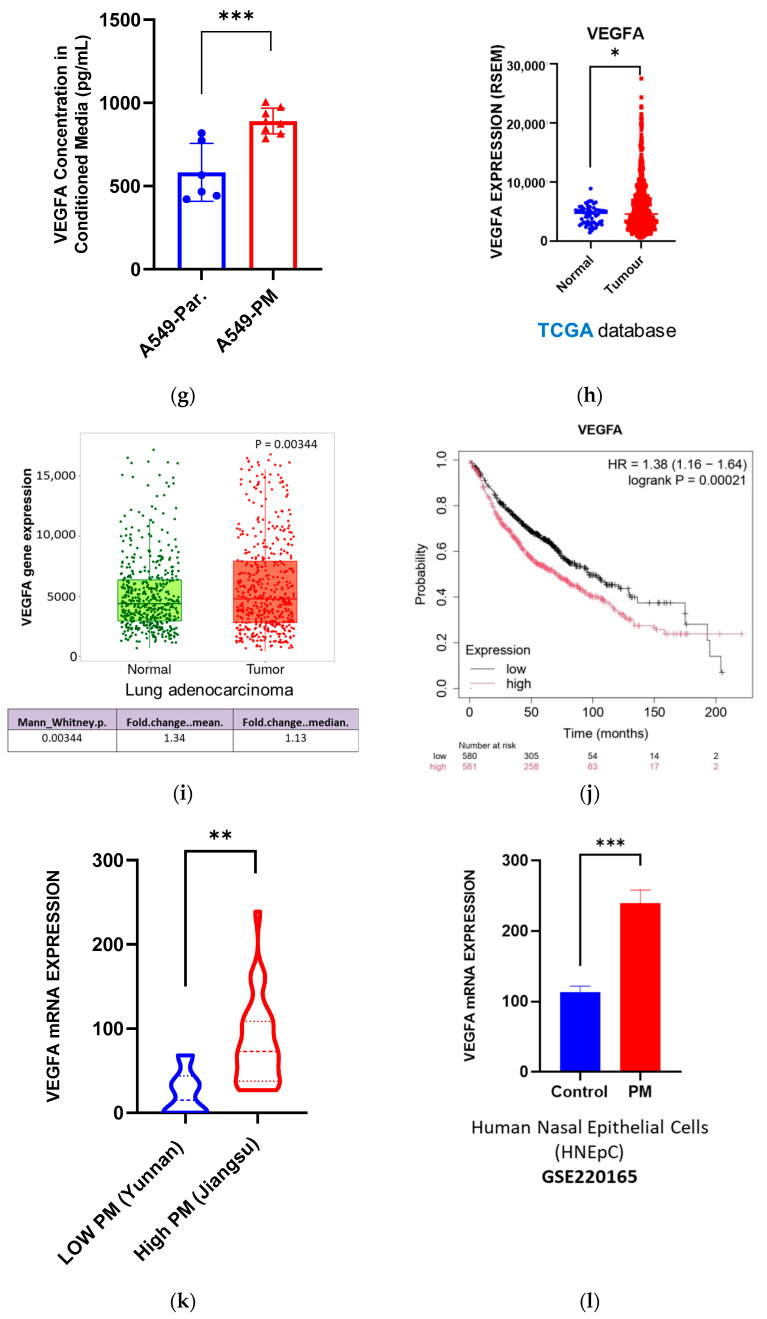
PM promotes angiogenesis in NSCLC by overexpression of VEGFA. (**a**) Pearson’s correlation analysis between PM atmospheric concentration and lung cancer mortality rate in 47 states in the USA. (**b**) Enrichment analysis of NSCLC tissues of female patients from Xuanwei city analysis performed by the TNM website (https://tnmplot.com/analysis/, accessed on 24 June 2024. Data source: Zhang Y, et al. (GSE89039). Green arrow points at sprouting angiogenesis. (**c**) mRNA expression of angiogenesis factors in A549-Par and A549-PM performed by q-PCR. (**d**) Protein expression of angiogenesis factors in A549-Par and A549-PM was performed by Western blot. (**e**) VEGFA expression difference between H1299-Par and H1299-PM. (**f**) VEGFA expression difference between LLC-Par and LLC-PM. (**g**) VEGFA concentration in conditioned media (CM) of A549-Par versus A549-PM. (**h**) VEGFA mRNA expression analysis of NSCLC tissues versus normal tissues generated from the TCGA database. (**i**) BoxPlot of VEGFA gene expression in lung cancer tissues versus normal. (**j**) TNM Kaplan–Meier survival analysis of NSCLC patients according to VEGFA expression in tumor tissues. (**k**) Difference in VEGFA mRNA expression in NSCLC tissues from provinces in China with low air concentration of PM (Yunnan: GSE165298) versus high concentration of PM (Jiangsu: GSE268175). (**l**) Deference in VEGFA mRNA expression between the human nasal epithelial cell line before and after PM exposure. (* for *p* < 0.05, ** for *p* < 0.01, *** for *p* < 0.001). Original, uncropped Western blot images show in Supplemental Materials.

A comprehensive analysis of data from 47 U.S. states revealed a strong positive correlation between the age-standardized lung cancer mortality rate (per 100,000 population, 95% uncertainty interval) and PM2.5 concentrations (Pearson correlation coefficient, r = 0.7638; 95% confidence interval: 0.6166–0.8594; R^2^ = 0.5834; *p* < 0.0001) (Figure 1a). The study utilized data from the Veterans Health Administration (VHA) databases and the Environmental Protection Agency’s (EPA) Community Multiscale Air Quality (CMAQ) Modeling System, as reported by Bowe et al. [15]. Relevant resources include the VHA Library Database (VA.gov) and the CDC Environmental Public Health Tracking Network (ephtracking.cdc.gov).

### 3.2. PM Promotes Angiogenesis in NSCLC

Previous studies have demonstrated that PM contributes to the development of lung cancer [40]. To explore whether PM promotes angiogenesis during lung cancer progression, we analyzed gene expression data from lung adenocarcinoma and adjacent normal tissues, as reported in a prior study [41], using TNMplot.com [42]. Gene Ontology Biological Processes (GO-BP) analysis revealed significant alterations in gene sets associated with sprouting angiogenesis (GO:0002040), highlighting a potential role of PM in promoting angiogenesis mechanisms during lung cancer progression (Figure 1b).

### 3.3. PM Long-Term Exposure Increases Vascular Endothelial Growth Factor A (VEGFA) Expression

A model of PM exposure cells were developed in Tang’s laboratory and Tan’s laboratory. A549 (human), H1299 (human) and LLC (mouse) lung adenocarcinoma cells (A549-Par, H1299-Par, and LLC-Par) were exposed to PM for a long-term period (30–60 days) to establish a PM-adapted cell line (A549-PM, H1299-PM, and LLC-PM). Tang’s lab studies have shown that the A549-PM cell line is an excellent model for in vitro and in vivo experiments for PM exposure study [37]. Therefore, we used these cells (A549-Par and A549-PM) and established two more cell lines in Tan’s laboratory (H1299-PM and LLC-PM) for our subsequent experiments.

To identify angiogenesis factors involved in lung adenocarcinoma progression, we conducted q-PCR and Western blot analyses to assess the mRNA and protein expression levels of a panel of angiogenesis factors. VEGFA was the only angiogenesis factor significantly overexpressed in A549-PM cells compared to A549-Par cells at both mRNA (Figure 1c) and protein levels (Figure 1d). Similarly, VEGFA was overexpressed in H1299-PM and LLC-PM cells compared to their corresponding parental cells (H1299-Par and LLC-Par), (Figure 1e,f). Consistent with these findings, ELISA results showed that VEGFA concentration in the conditioned media of A549-PM cells was significantly higher than that of A549-Par cells (Figure 1g).

To explore the clinical relevance of VEGFA in lung cancer progression, we analyzed data from The Cancer Genome Atlas (TCGA) and TNMplot.com. VEGFA mRNA expression was significantly elevated in lung adenocarcinoma tissues compared to adjacent normal tissues (Figure 1h,i). Additionally, Kaplan–Meier survival analysis of a cohort of 2166 patients demonstrated that high VEGFA levels were significantly associated with poorer prognosis in NSCLC patients compared to lower VEGFA levels (Figure 1j). These findings suggest that VEGFA overexpression plays a critical role in disease progression and is a negative prognostic factor in lung cancer.

To investigate further, we analyzed data from NSCLC patients residing in different provinces in China with high (Jiangsu) or low (Yunnan) ambient PM concentrations. Tumor tissues from high-PM areas exhibited significantly higher levels of VEGFA gene expression compared to those from low-PM areas (Figure 1k) ([41], [GSE165298]; [43] [GSE268175]). Further supporting these findings, analyzing a dataset of human nasal epithelial cells (HNEpC) exhibited a significant increase in VEGFA mRNA expression following PM exposure compared to pre-exposure levels (Figure 1l). Taken together, these results demonstrate that PM exposure promotes VEGFA expression in lung cancer cells.

### 3.4. PM Exposure Promotes Angiogenesis Through Up-Regulation of VEGFA In Vitro

To investigate the effects of VEGFA in PM-promoted angiogenesis, we performed the in vitro angiogenesis assays. Conditioned media (CM) obtained from A549-Par and A549-PM cells and endothelial progenitor cells (EPCs) were used to conduct the tube formation and Boyden chamber migration assays. EPCs were incubated with the following media: CM from A549-Par cells, CM from A549-PM cells, CM from A549-Par supplemented with VEGFA recombinant protein (positive control), CM from A549-PM supplemented with VEGFA-neutralizing antibody, or CM from A549-PM supplemented with IgG.

**Figure 2:**
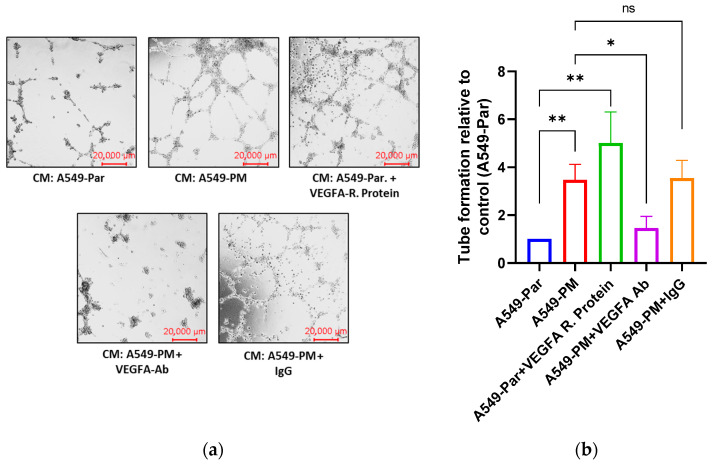

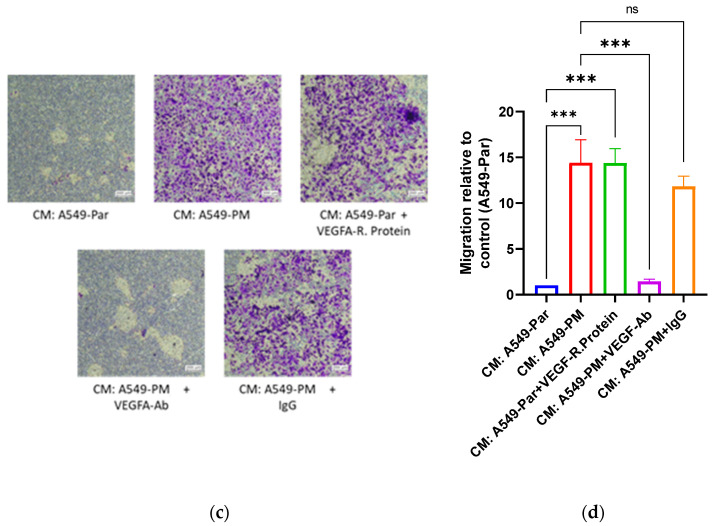
PM-led overexpression of VEGFA promotes EPCs tube formation and migration. (**a**,**b**) Tube formation by EPCs exposed to CM of A549-Par, CM of A549-PM, CM of A549-Parental + VEGFA-recombinant protein (positive control), CM of A549-PM + VEGFA neutralizing antibody, or CM of A549-PM + IgG antibody (negative control). (**c**,**d**) Migration of EPCs test performed by Boyden chamber after incubating EPCs with CM of A549-Par, CM of A549-PM, CM of A549-Parental + VEGFA-recombinant protein (positive control), CM of A549-PM + VEGFA neutralizing antibody, or CM of A549-PM + IgG antibody (negative control). Quantification was performed for tube formation and migration tests utilizing ImageJ software. (* for *p* < 0.05, ** for *p* < 0.01, *** for *p* < 0.001, ns for not significant).

In the tube formation assay, EPCs incubated with CM from A549-PM showed significantly greater tube formation compared to those treated with CM from A549-Par. Similarly, adding VEGFA recombinant protein to the CM from A549-Par significantly enhanced tube formation. Conversely, supplementation of VEGFA-neutralizing antibody with CM from A549-PM significantly inhibited tube formation. However, adding IgG to CM from A549-PM had no significant effect, with results comparable to EPCs treated with CM from A549-PM alone (Figure 2a). Quantification of tube formation was performed using ImageJ software version 1.x (Figure 2b).

Consistent with the tube formation findings, EPCs exhibited significantly greater migration when treated with CM from A549-PM compared to CM from A549-Par in the Boyden chamber migration assay (Figure 2c,d). These results indicate that PM exposure promotes angiogenesis, as evidenced by enhanced EPCs tube formation and migration, primarily through the overexpression of VEGFA.

### 3.5. PM Promotes VEGFA Overexpression via the MAPK Pathway

**Figure 3:**
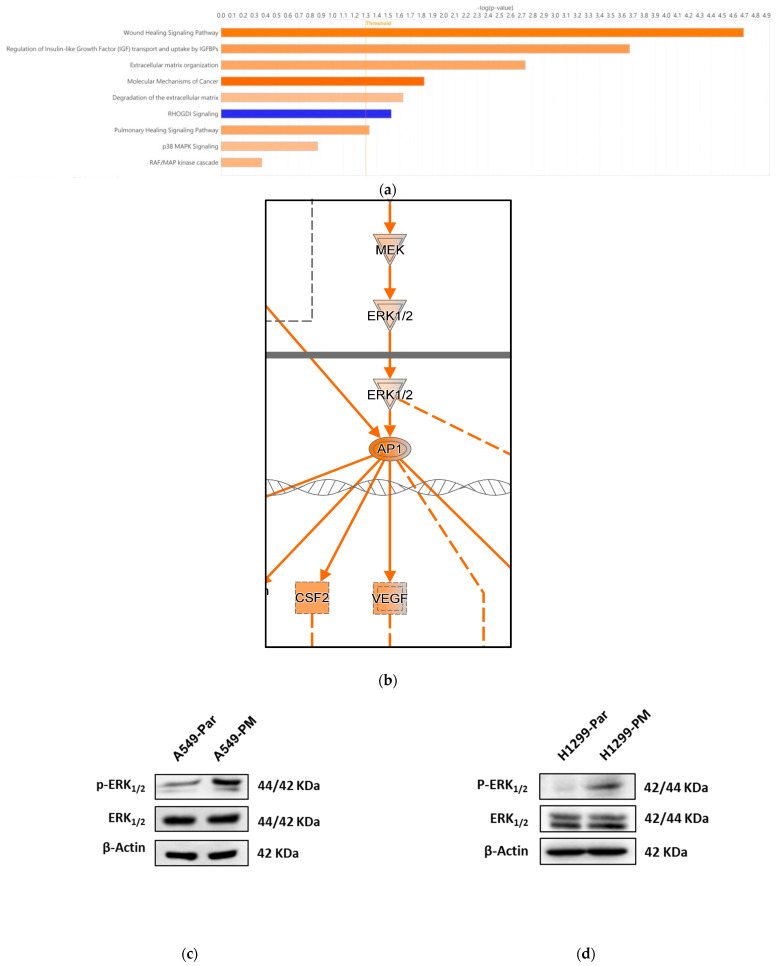

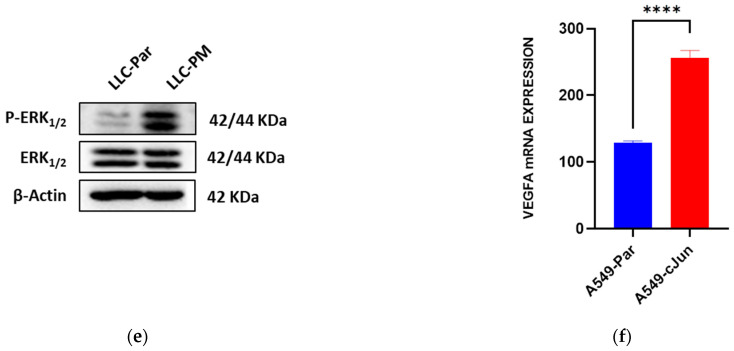
PM promotes VEGFA overexpression through the MAPK pathway. (**a**) Ingenuity pathway analysis (IPA) of the RNA-seq gene expression shows the functional analysis of A549-Par and A549-PM. (**b**) Wound healing signaling pathway as identified by IPA. (**c**–**e**) Western blot analysis of p-ERK1/2 in A549-PM, H1299-PM, and LLC-PM. (**f**) Relative VEGFA mRNA expression between parental A549 (A549-Par) and c-Jun overexpression plasmid transfected A549 (A549-cJun) (GSE213590). (**** for *p* < 0.0001). Original, uncropped Western blot images show in Supplemental Materials.

To investigate the mechanism underlying VEGFA overexpression in A549-PM cells, we performed an RNA-seq of A549-Par and A549-PM [37], and we analyzed the data using Ingenuity Pathway Analysis (IPA), a bioinformatics tool for analyzing and interpreting gene expression data (www.ingenuity.com). The results indicated that the wound healing signaling pathway was the most enriched pathway in A549-PM cells (Figure 3a). Further analysis of this pathway suggested that the mitogen-activated protein kinase (MAPK) pathway might play a role in VEGFA overexpression (Figure 3b).

To clarify which pathway is primarily involved, Western blot analysis was conducted. The results showed that phosphorylated extracellular signal-regulated kinase (p-ERK) protein expression was significantly higher in A549-PM, H1299-PM, and LLC-PM cells compared to their corresponding parental cells, indicating activation of the MAPK/ERK pathway (Figure 3c–e).

Since it is well known that the AP-1 transcription factor complex, which comprises c-Jun and c-Fos, is downstream of the MAPK/ERK pathway, we analyzed a dataset (GSE213590, available at ncbi.nlm.nih.gov) that was from the control and A549 cells transfected with c-Jun. As expected, A549 cells with a c-Jun overexpression, which mimics MAPK/ERK activation, showed a significant increase in VEGFA mRNA levels compared to pre-transfection levels (Figure 3f). This observation aligns with the IPA analysis of the wound healing signaling pathway, which identified the AP-1 transcription factor complex (c-Jun and c-Fos) as a potential regulator of VEGFA overexpression. These findings suggest that PM exposure promotes VEGFA overexpression in LUAD cells predominantly through the activation of the MAPK/ERK pathway and the involvement of AP-1 transcription factors.

### 3.6. Inhibition of the MAPK Pathway Blocks PM-Enhanced VEGFA Expression and Angiogenesis Properties

**Figure 4:**
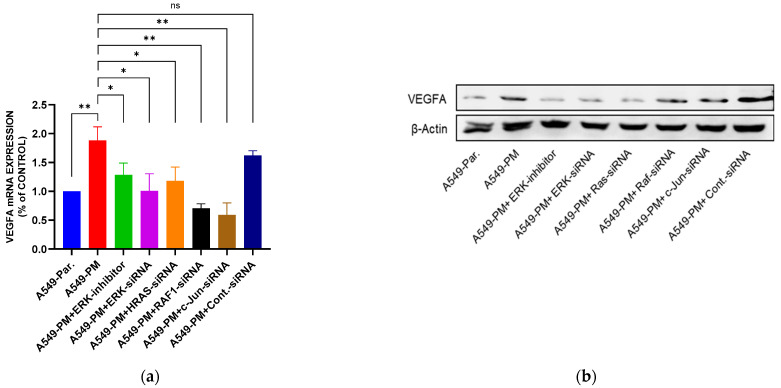

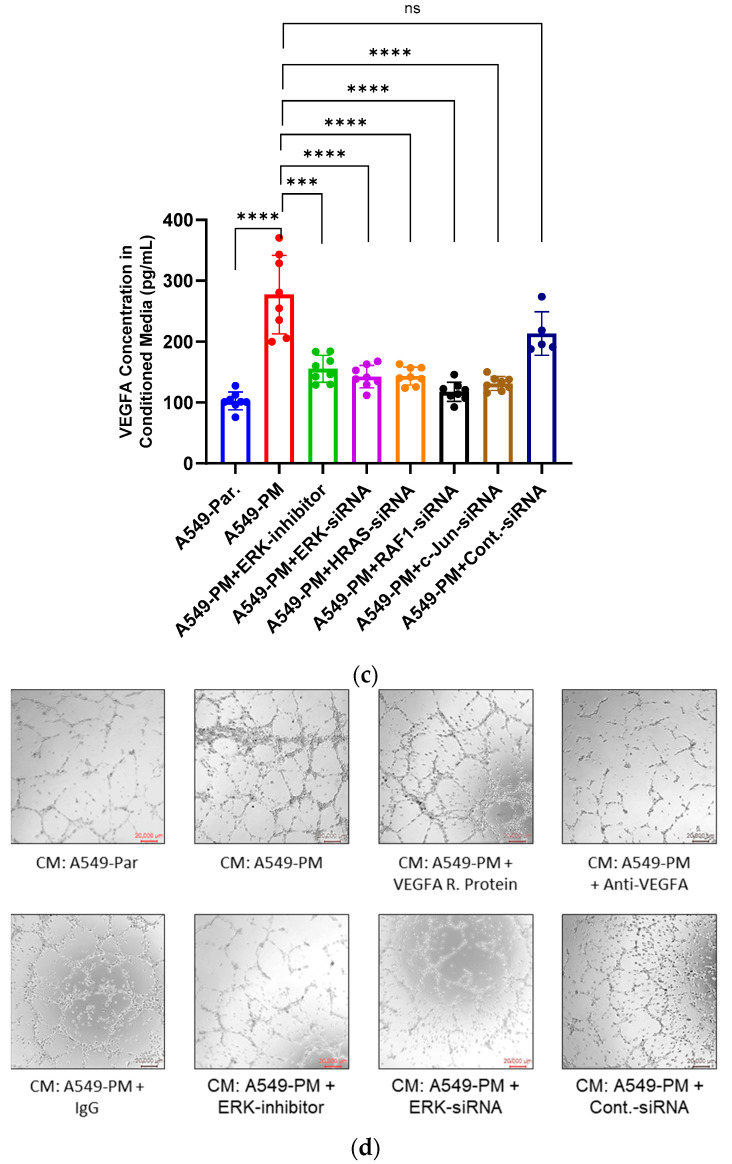

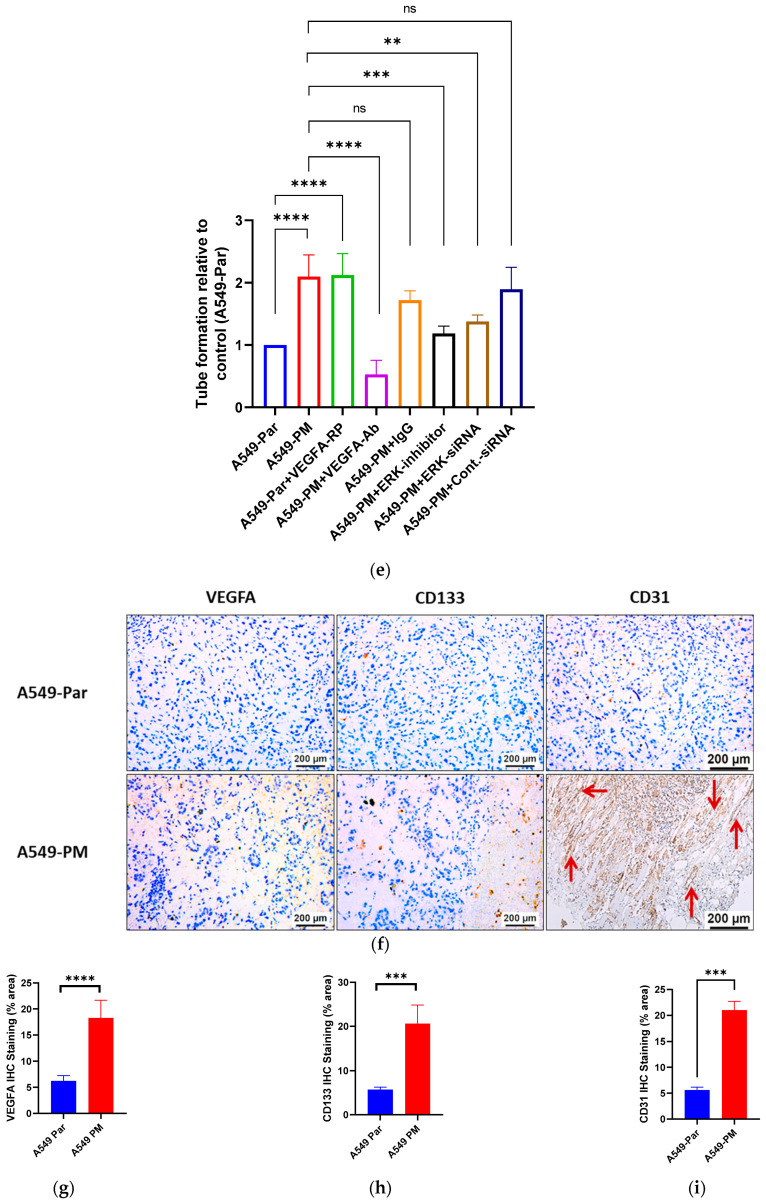
PM promotes VEGFA overexpression through the MAPK pathway in the A549-PM cell line. A549-PM cells were treated with siRNA of different proteins of the MAPK pathway. (**a**) Cells were treated as indicated and expression of VEGFA mRNA was verified by qPCR. (**b**) Western blot shows VEGFA expression in differently treated cells. (**c**,**d**) Tube formation by EPCs treated with different CMs is shown with quantification of tube formation using ImageJ software. (**e**) ELISA result of VEGFA concentration in CM after treatment as indicated. (**f**–**i**) Tissues from xenograft tumors obtained from sacrificed mice and IHC-stained with VEGFA, CD133, and CD31. Red arrows are pointing to blood vessels. (* for *p* < 0.05, ** for *p* < 0.01, *** for *p* < 0.001, **** for *p* < 0.0001, ns for not significant). Original, uncropped Western blot images show in Supplemental Materials.

To confirm the role of the MAPK pathway in VEGFA overexpression in A549-PM cells, we utilized short interfering RNA (siRNA) to silence specific MAPK pathway genes. The results demonstrated that silencing these genes significantly reduced both VEGFA mRNA and protein expression levels, as shown in Figure 4a,b. Consistent with these findings, VEGFA concentrations in the conditioned media (CM) of MAPK-silenced A549-PM cells were markedly decreased, as presented by ELISA analysis (Figure 4c).

To further investigate whether MAPK inhibition affects angiogenesis, we conducted a tube formation assay using CM from A549-PM cells treated with either an ERK inhibitor or ERK-specific siRNA. The results showed that both ERK inhibitor and ERK-specific siRNA blocked PM-promoted tube formation by EPCs, highlighting the anti-angiogenesis effects of MAPK pathway inhibition (Figure 4d,e).

These findings collectively indicate that the MAPK pathway plays a critical role in VEGFA overexpression and angiogenesis in A549-PM cells.

### 3.7. PM Promotes Angiogenesis in an In Vivo Study via VEGFA Overexpression

To validate our in vitro findings, we performed an immunohistochemistry staining of tumor tissues from a previous animal study [37]. The results demonstrated a significant overexpression of VEGFA, CD31 (a vascular marker), and CD133 (a marker for human hematopoietic precursor and EPC cells) in A549-PM xenograft tumor tissues compared to A549-Par tissues (Figure 4f–i). Tissues stained with CD31 displayed extensive vascularization, with multiple blood vessel linings observed across the slides on A549-PM tissues (illustrated by red arrows, Figure 4f).

## 4. Discussion

Air pollution is explicitly addressed in two WHO’s SDG targets: SDG 3.9, which focuses on significantly reducing health risks from hazardous substances, and SDG 11.6, which aims to minimize the negative impacts of cities on people. This study provides different insights into the mechanistic link between ambient PM exposure and lung cancer progression, with a particular focus on angiogenesis and the overexpression of VEGFA. Through a combination of epidemiological, in vitro, and in vivo analyses, our findings underscore the critical role of PM in promoting tumor angiogenesis and elucidate the underlying molecular pathways, thereby advancing our understanding of the adverse health effects of air pollution.

### 4.1. Elevated PM Levels and Lung Cancer Mortality

The strong positive correlation between PM concentrations and lung cancer mortality rates observed across 47 U.S. states highlights the significant public health burden posed by air pollution. These findings are consistent with previous studies showing that long-term exposure to elevated PM levels increases lung cancer incidence and mortality [12,44,45,46,47]. The underlying mechanisms likely involve PM-induced genetic mutations, oxidative stress, and inflammation [33,48,49,50,51,52,53]. In our analysis, the high Pearson correlation coefficient (r = 0.7638) and robust statistical significance (*p* < 0.0001) emphasize the strength of this association, underscoring the need for stringent air quality regulations to mitigate adverse health outcomes (Figure 1a). Notably, the mortality burden attributable to ambient PM varies significantly across global regions. For instance, the Asian domain reports the highest per-capita mortality rate attributed to PM (63 deaths per 100,000 population; 2.3 million deaths), whereas the northern Americas domain reports the lowest (25 deaths per 100,000 population; 150,000 total deaths) [11]. These findings highlight regional disparities and the urgent need for targeted interventions to address this pressing global health challenge.

### 4.2. PM-Induced Angiogenesis in NSCLC

Our findings provide compelling evidence that long-term exposure to PM promotes angiogenesis in NSCLC. Gene Ontology enrichment analysis of transcriptomic data from female NSCLC patients in Xuanwei City, China, an area known for extremely high indoor PM exposure, revealed significant up-regulation of gene sets associated with sprouting angiogenesis in LUAD tissues. Notably, Xuanwei, a rural region in southwestern China, exhibits the highest incidence and mortality rates of lung cancer in the country. Women in this area, who are predominantly non-smokers (smoking prevalence < 1%), experience the highest rates of lung cancer among females in China, with incidence peaking at a younger age (41–50 years). This high disease burden has been strongly linked to the domestic use of smoky coal, which results in exceptionally elevated indoor PM concentrations reaching up to 24.4 mg/m^3^ [41]. In contrast, the recommended indoor PM2.5 standard set by the American Society of Heating, Refrigerating and Air-Conditioning Engineers (ASHRAE) Standards 62.1 and 62.2 is <15 µg/m^3^ [54]. Furthermore, VEGFA was identified as a key angiogenesis factor overexpressed in PM-adapted lung cancer cell lines (A549-PM, H1299-PM, and LLC-PM). The critical role of VEGFA in tumor angiogenesis was further validated by its association with poorer prognosis in NSCLC patients, as revealed by Kaplan–Meier survival analysis involving 2166 lung cancer patients (kmplot.com). Additionally, our data revealed that VEGFA mRNA expression in NSCLC patients from China who share the same Asian ancestry varies based on their region of residence. Specifically, VEGFA gene expression is significantly higher in NSCLC tissues from patients living in areas with high atmospheric PM concentrations compared to those residing in areas with lower concentrations. The analysis of normal bronchial epithelial cells demonstrated that PM significantly up-regulates VEGFA mRNA expression, aligning with previous studies that revealed the ability of PM to promote inflammation and angiogenesis in normal bronchial epithelial tissue [55]. The latter findings suggest that angiogenesis may be a critical step in the malignant transformation of normal bronchial epithelial cells.

Consequently, the functional significance of VEGFA in PM-induced angiogenesis was demonstrated through tube formation and migration assays. Conditioned media (CM) from A549-PM cells enhanced both tube formation and migration of EPCs, effects that were reversed by VEGFA-neutralizing antibodies. These results further support the role of VEGFA as a central mediator of tumor angiogenesis in the context of air pollution.

### 4.3. Molecular Mechanisms Underlying VEGFA Overexpression

Our study elucidates the molecular mechanisms underlying VEGFA overexpression induced by PM exposure. Ingenuity Pathway Analysis (IPA) identified the MAPK/ERK pathway as a primary regulator of VEGFA expression in A549-PM cells via regulation of AP-1 transcription factor complex, which is a dimeric transcription factor that consists of members of the Fos and Jun gene families [56,57]. Western blot analyses confirmed elevated phosphorylated ERK levels in A549-PM, H1299-PM, and LLC-PM cells. Additionally, data from an independent source revealed increased VEGFA gene expression in c-Jun-overexpressing A549 cells, further supporting the role of the AP-1 transcription factor complex in this process. Importantly, inhibition of the MAPK pathway significantly reduced VEGFA expression and angiogenesis, as evidenced by decreased tube formation in EPCs. These findings suggest that targeting the MAPK/ERK pathway may represent a promising therapeutic strategy for mitigating PM-induced angiogenesis in lung cancer. It would be interesting to explore combining clinically approved agents, such as trametinib (a MEK inhibitor) and bevacizumab (an anti-VEGF monoclonal antibody), for potential repurposing in PM-associated lung cancer treatment.

It is noteworthy that Ji SM and colleagues demonstrated VEGFA overexpression in A549 cells following exposure to PM in vitro. They further reported that the increase in angiogenesis activity was mediated by hypoxia-inducible factor-1α (HIF-1α) [32]. However, their study only applied low PM concentrations (1 and 10 μg/mL) with a short exposure duration of 24 h. Notably, it was previously reported that NSCLC and non-cancerous bronchial epithelial cell lines react differently to different exposure durations of PM [36,58,59,60]. These defects seriously limited the clinical relevance of Ji SM’s study. In our study, we aimed to investigate the effects of long-term PM exposure at a concentration between 12.5 and 25 μg/mL on NSCLCs. This approach aligns with previous studies on the effects of PM on cell lines while more reasonably reflecting real-world pollution exposure experienced by lung cancer patients, thereby providing deeper insights into its impact on disease progression [37,60,61].

### 4.4. In Vivo Validation of PM-Induced Angiogenesis

Our in vivo studies provide additional validation of the pro-angiogenesis effects of PM exposure. Immunohistochemical analyses of A549-Par and A549PM xenograft tumor tissues revealed significant overexpression of VEGFA and vascular markers such as CD31 and CD133 in A549-PM-derived tumors. Extensive vascularization observed in these tissues underscores the role of PM in enhancing tumor angiogenesis and progression. Data about differences in tumor growth, size, and weight between A549-Par and A549-PM tumor tissues of the same mice were presented in a previous study, where A549-PM tissues exhibited significantly greater tumor growth, weight, and volume compared to A549-Par tissues [37].

While our study provides comprehensive evidence connecting PM exposure to lung cancer progression, certain limitations should be acknowledged. Our in vitro and in vivo models may not fully reproduce the complex interactions between PM and the tumor microenvironment in human populations. Notably, the main in vitro model of this study is the A549 cell line, which harbors driver mutations such as KRAS and KEAP1, which can affect VEGFA expression and angiogenesis. However, since both parental and PM-exposed cells share the same genetic background, the observed VEGFA overexpression can be attributed solely to PM exposure. Consequently, findings derived from this model may not fully recapitulate the heterogeneity of LUAD in vivo and warrant caution in extrapolating results to broader clinical contexts. In addition, our in vitro analyses focused primarily on MAPK/ERK/VEGFA axis; other angiogenesis-related factors and pathways involved in the upregulation of VEGFA may also play important roles and warrant further investigation. Finally, regional variations in the composition of PM and associated health effects were not explored in detail, which could influence the generalizability of our findings.

## 5. Conclusions

In conclusion, the findings of this study have significant implications for public health and lung cancer therapeutics. Given the robust association between PM exposure and VEGFA overexpression, efforts to reduce ambient air pollution could play a critical role in decreasing lung cancer incidence and mortality. Additionally, the identification of the MAPK/ERK pathway as a key driver of angiogenesis induced by PM provides a potential target for therapeutic intervention. Future studies should explore the efficacy of MAPK inhibitors in clinical settings and investigate the role of other pathways that may contribute to carcinogenesis mediated by PM.

## Figures and Tables

**Table 1:** Different primers used in this study.

	Forward (5′–3′)	Reverse (5′–3′)
VEGFA	GCAGA-ATCAT-CACGA-AGTGG	GCATG-GTGAT-GTTGG-ACTCC
VEGFB	GAGATGTCCCTGGAAGAACACA	GAGTGGGATGGGTGATGTCAG
ANGPIT1	CAAGGCCATCTGTGAAAACAAG	CAGGGGGAACCTCCATGTAG
FGF1	GCCCTGACCGAGAAGTTTAATC	CCCCGTTGCTACAGTAGAGG
FGF13	AGGCCGAGGGTGGTATCTG	AGATCGGGAGAACTCCGTGAG
FGF22	GGGAGCGCATCGAAGAGAAC	CTGTGAGGCGTAGGTGTTGTG
PDGFB	CTCGATCCGCTCCTTTGATGA	CGTTGGTGCGGTCTATGAG

## Data Availability

All data sources are provided within the text, references, or legends of the figures.

## Supplementary Materials

The following supporting information can be downloaded at: https://www.mdpi.com/article/10.3390/cancers17172868/s1, Original, uncropped Western blot images.
