# Supplementary material for: Long-Term Particulate Matter (PM) Exposure Promotes Non-Small-Cell Lung Cancer (NSCLC) Angiogenesis Through Up-Regulation of VEGFA
Source: Cancers (Basel). 2025 Aug 31;17(17):2868. doi: 10.3390/cancers17172868 (PMC12427299; doi:10.3390/cancers17172868)
Supplement: Supplementary file 1 [file cancers-17-02868-s001.zip › cancers-3748904-supplementary.pdf]

Figure 1d

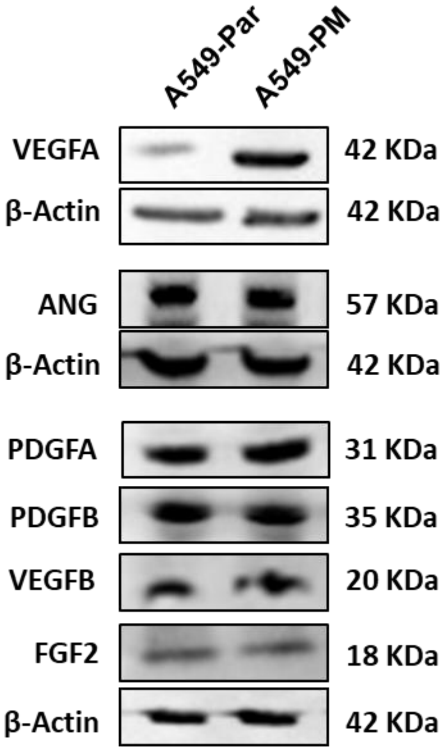
A549-Par
A549-PM
VEGFA
42 KDa
β-Actin
42 KDa
ANG
57 KDa
β-Actin
42 KDa
PDGFA
31 KDa
PDGFB
35 KDa
VEGFB
20 KDa
FGF2
18 KDa
β-Actin
42 KDa

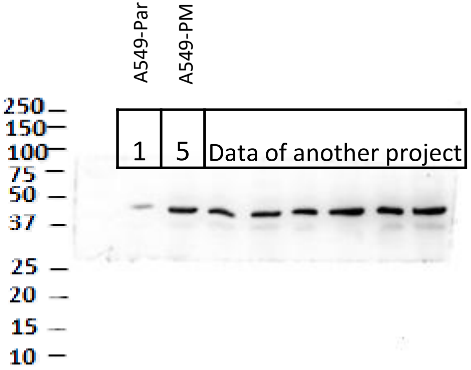

VEGFA

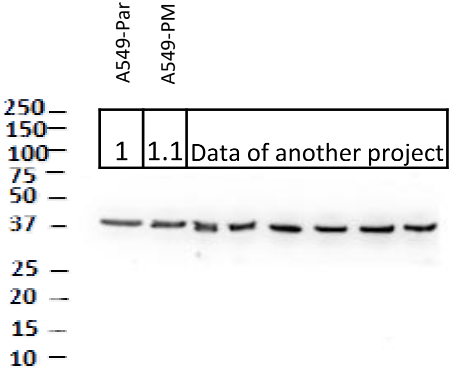

B-Actin

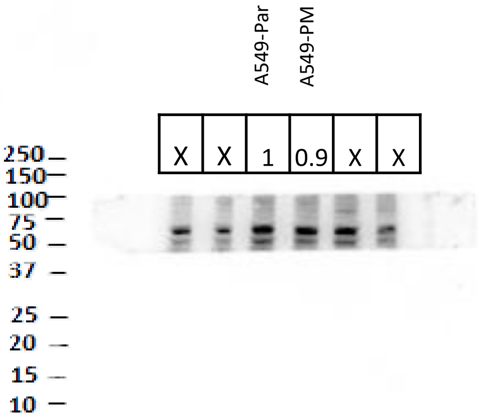

ANG

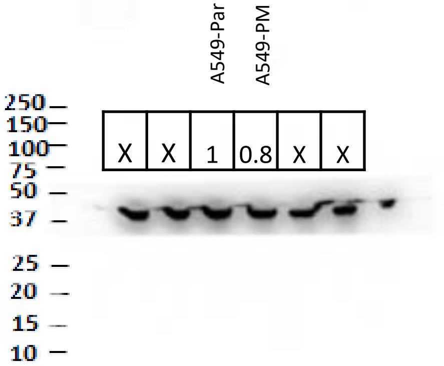

B-Actin

X = Data of another project

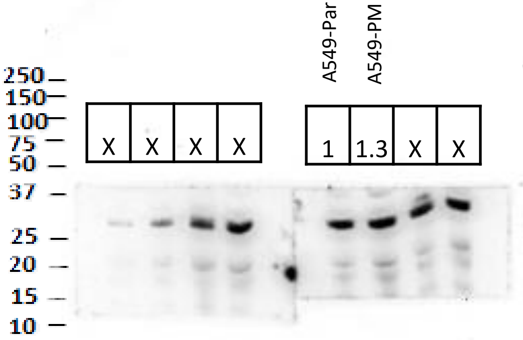

PDGFA

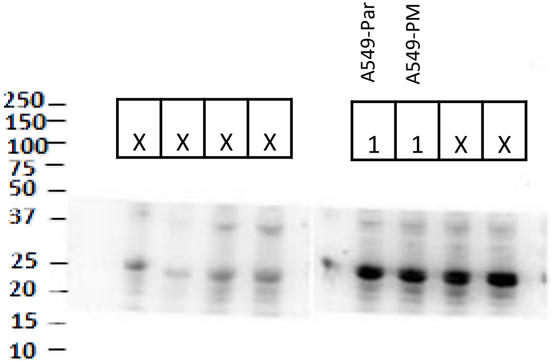

PDGFB

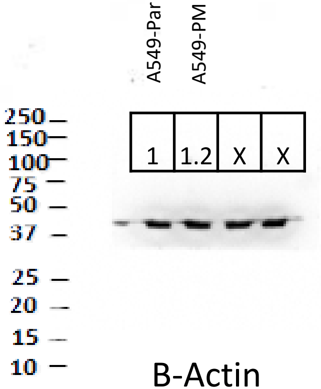

B-Actin

X = Data of another project

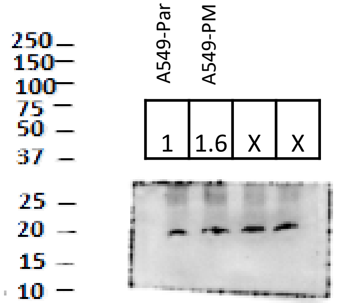

VEGFB

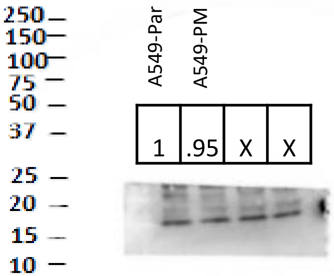

FGF2

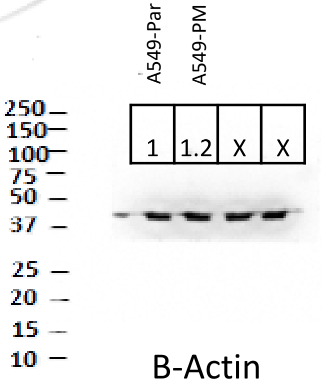

B-Actin

X = Data of another project

Figure 1e

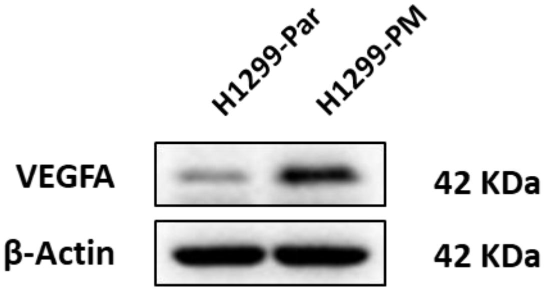
H1299-Par
H1299-PM
VEGFA
β-Actin
42 KDa
42 KDa

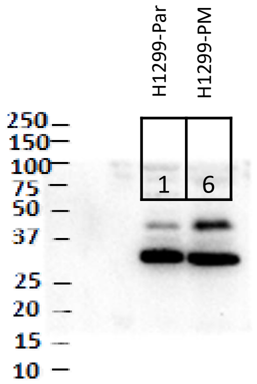

VEGFA

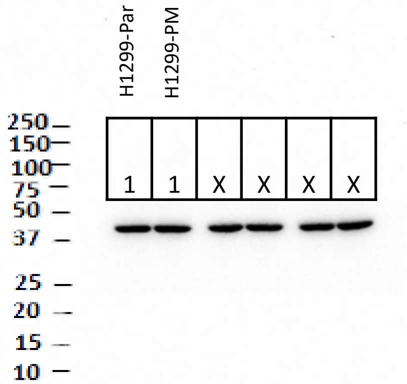

B-Actin

Figure 1f

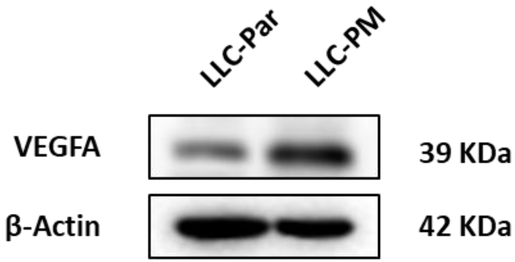
LLC-Par
LLC-PM
VEGFA
39 KDa
β-Actin
42 KDa

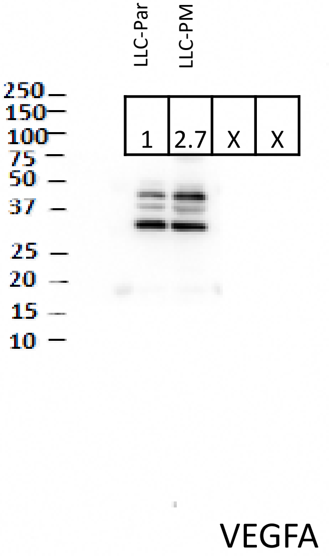
LLC-Par
LLC-PM
250
150
100
75
50
37
25
20
15
10
1
2.7
X
X
VEGFA

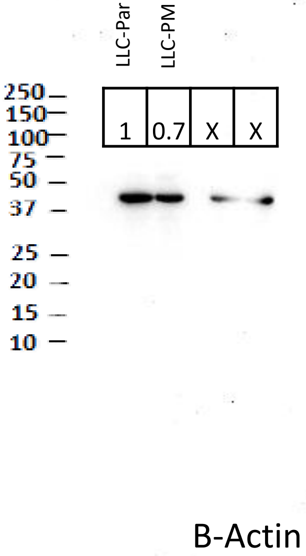
LLC-Par
LLC-PM
250
150
100
75
50
37
25
20
15
10
1
0.7
X
X
B-Actin

Figure 3c

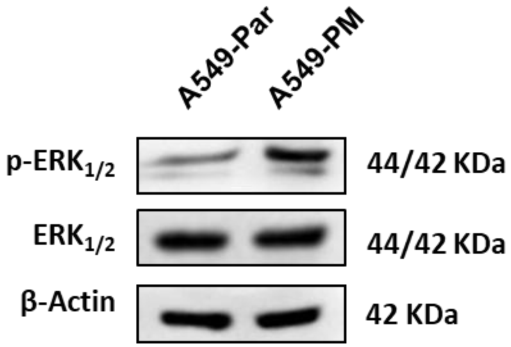
A549-Par
A549-PM
p-ERK1/2
ERK1/2
β-Actin
44/42 KDa
44/42 KDa
42 KDa

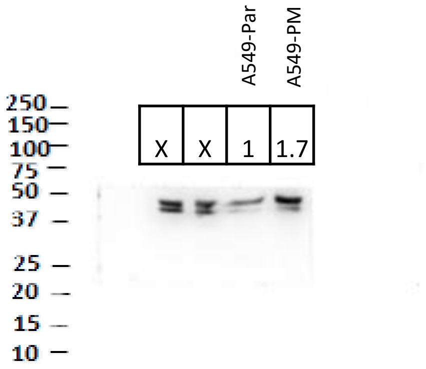

p-ERK

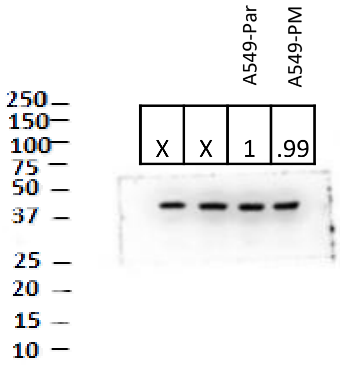

ERK

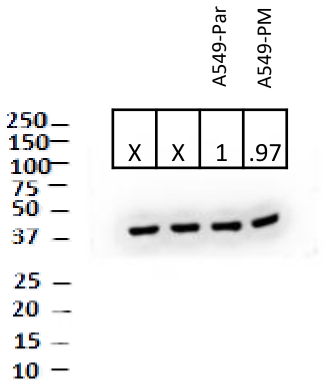

B-Actin

X = Data of another project

Figure 3d

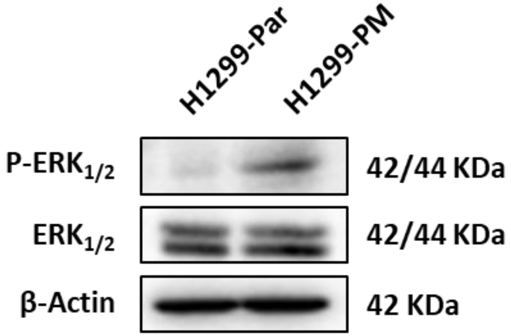
H1299-Par
H1299-PM
P-ERK1/2
42/44 KDa
ERK1/2
42/44 KDa
β-Actin
42 KDa

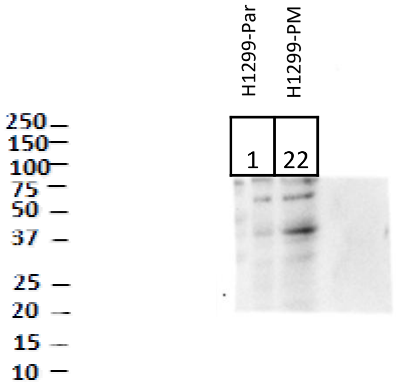

p-ERK

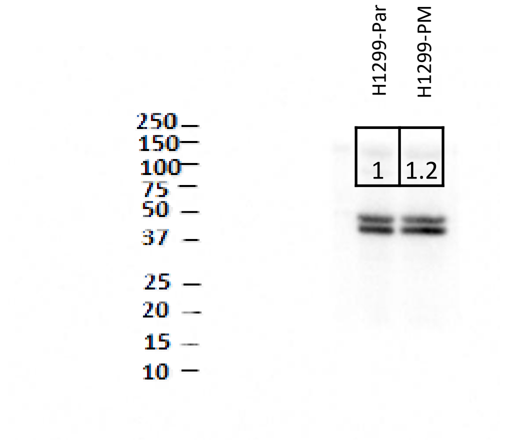

ERK

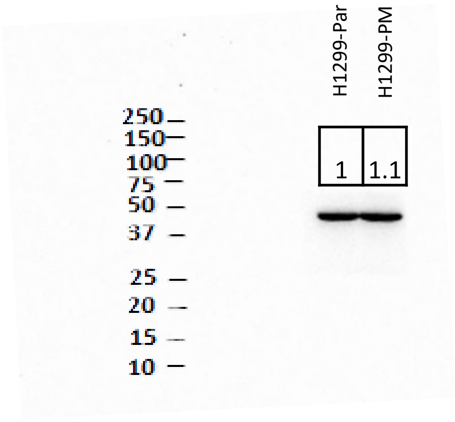

B-Actin

X = Data of another project

Figure 3e

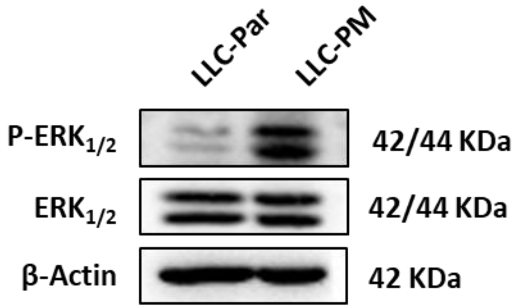
LLC-Par
LLC-PM
P-ERK1/2
ERK1/2
β-Actin
42/44 KDa
42/44 KDa
42 KDa

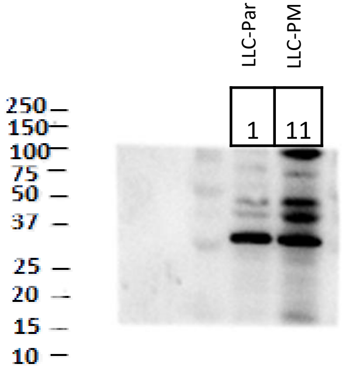

p-ERK

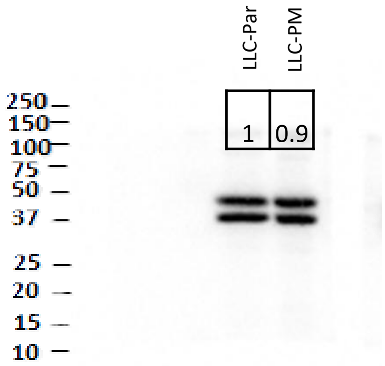

ERK

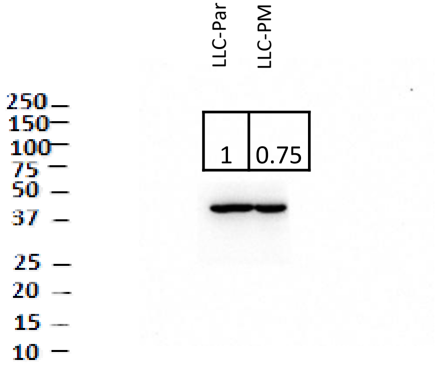

B-Actin

X = Data of another project

Figure 4b

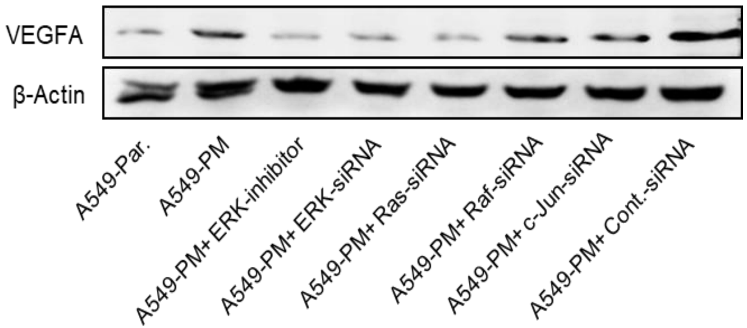
VEGFA
β-Actin
A549-Par.
A549-PM
A549-PM+ ERK-inhibitor
A549-PM+ ERK-siRNA
A549-PM+ Ras-siRNA
A549-PM+ Raf-siRNA
A549-PM+ c-Jun-siRNA
A549-PM+ Cont.-siRNA

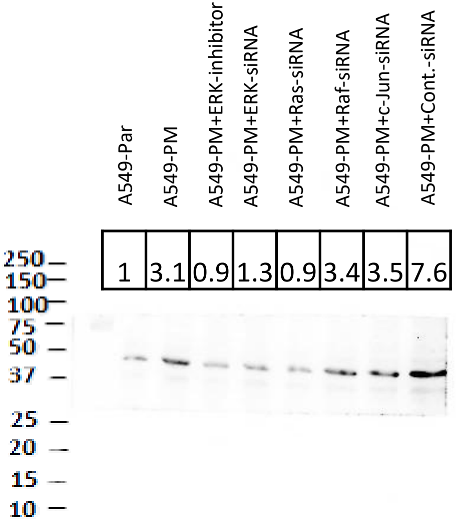

VEGFA

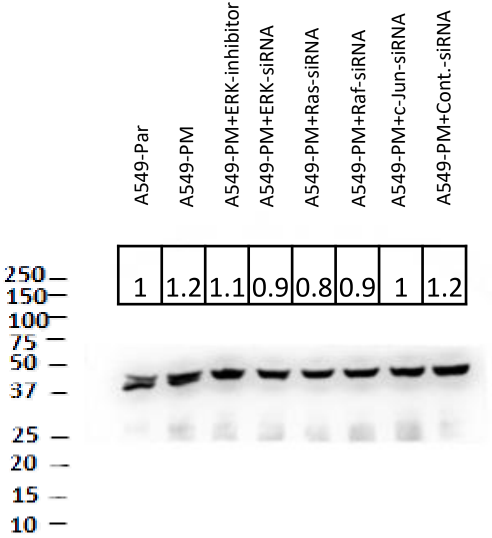

B-Actin
